# Integration of Family Planning Services into HIV Care and Treatment Services: A Systematic Review

**DOI:** 10.1111/sifp.12018

**Published:** 2017-03-24

**Authors:** Sabina A. Haberlen, Manjulaa Narasimhan, Laura K. Beres, Caitlin E. Kennedy

## Abstract

Evidence on the feasibility, effectiveness, and cost‐effectiveness of integrating family planning (FP) and HIV services has grown significantly since the 2004 Glion Call to Action. This systematic review adds to the knowledge base by characterizing the range of models used to integrate FP into HIV care and treatment, and synthesizing the evidence on integration outcomes among women living with HIV. Fourteen studies met our inclusion criteria, eight of which were published after the last systematic review on the topic in 2013. Overall, integration was associated with higher modern method contraceptive prevalence and knowledge, although there was insufficient evidence to evaluate its effects on unintended pregnancy or achieving safe and healthy pregnancy. Evidence for change in unmet need for FP was limited, although two of the three evaluations that measured unmet need suggested possible improvements associated with integrated services. However, improving access to FP services through integration was not always sufficient to increase the use of more effective (noncondom) modern methods among women who wanted to prevent pregnancy. Integration efforts, particularly in contexts where contraceptive use is low, must address community‐wide and HIV‐specific barriers to using effective FP methods alongside improving access to information, commodities, and services within routine HIV care.

Like all women of reproductive age, women living with HIV have diverse fertility intentions that change over time and are influenced by interrelated factors at the individual, couple, family, and community levels. Moreover, the sexual and reproductive health and rights (SRHR) of women living with HIV are further influenced by factors such as stigma and discrimination, gender inequality, violence, lack of community empowerment, coercion and lack of informed choice from health care providers, and unsupportive laws and policies (Nattabi et al. 2009; MacCarthy et al. 2012; WHO 2012). Access to antiretroviral treatment (ART) and comprehensive programs to eliminate perinatal transmission of HIV and syphilis as well as accurate, up‐to‐date information on HIV, contraception, safer conception, pregnancy, and HIV transmission are important programmatic factors shaping fertility desires and reproductive choices for women living with HIV (Kanniappan, Jeyapaul, and Kalyanwala 2008; Salamander Trust 2014).

Sub‐Saharan Africa, the region with the highest HIV prevalence, also has the highest prevalence of unmet need for contraception, where one in five women has unmet need for spacing or limiting pregnancies (UNFPA 2016). The problems associated with unmet need for contraception and unintended pregnancy are not limited by region. The 2014 global survey on the SRHR priorities of women living with HIV, the largest to date, led by and conducted among women living with HIV, found that 60 percent of respondents had at least one unplanned pregnancy and that less than half had ever obtained family planning (FP) services (Salamander Trust 2014). A cohort study in Johannesburg, South Africa reported that nearly one in four women had at least one unplanned pregnancy within two years of initiating ART and that 62 percent of the pregnancies were unplanned (Schwartz et al. 2012). The consequences of unintended pregnancies can be profound; safe abortion is not universally available, and, without optimal treatment, women living with HIV are at greater risk of death during the pregnancy and postpartum period than women without HIV (Calvert and Ronsmans 2013).

Family planning for women living with HIV has the dual goals of preventing unintended pregnancies and facilitating safe and healthy pregnancy among women who want to become pregnant. Enabling women and couples living with HIV to prevent unintended pregnancies and to plan for safe and healthy pregnancies will also prevent perinatal transmission of HIV (Halperin, Stover, and Reynolds 2009; Inter‐Agency Task Team for the Prevention and Treatment of HIV Infection in Pregnant Women and their Children 2012; Wilcher, Petruney, and Cates 2013). A package of essential FP services for women living with HIV at the community and health‐facility level has been developed that includes 1) information and counseling to support the right to make reproductive decisions, including pregnancy or prevention of unintended pregnancy, 2) effective clinical management of HIV to improve health irrespective of pregnancy intention, 3) rights‐based FP counseling and services, including contraceptives and infertility screening and services, 4) STI screening and management, 5) gender‐based violence (GBV) prevention and impact mitigation, and 6) an environment without stigma and discrimination (Inter‐Agency Task Team for the Prevention and Treatment of HIV Infection in Pregnant Women and their Children 2012). The inclusion of both GBV prevention and the elimination of stigma as essential components of FP services acknowledges that strengthening service access or quality may not suffice to improve health outcomes in the absence of a safe and supportive environment. Integrating these FP services with HIV services has been one approach to make both services more accessible to women and couples living with HIV. Since the Glion Call to Action linking FP and HIV (UNFPA and WHO 2004), significant progress has been made toward the integration of FP and HIV at multiple levels, from advocacy, policy planning, and funding to the integrated delivery of services in health facilities and communities (WHO, UNFPA, IPPF, and UNAIDS 2005; Farrell 2007; Wilcher et al. 2013; Medley et al. 2015).

Likewise, the evidence base on the feasibility, outcomes, and cost‐effectiveness of integrating FP and HIV services has grown significantly over the past decade. The first systematic review on integrated FP/HIV services included 16 studies, one of which integrated FP services within HIV care and treatment (Spaulding et al. 2009). The review demonstrated the feasibility of FP/HIV integration and its association with generally positive outcomes, but identified the need for more data on its effects on unintended pregnancies and cost‐effectiveness, as well as the reporting of process outcomes to document fidelity and identify factors that enhanced or hindered implementation. The most recent systematic review on FP/HIV integration included evidence from 12 studies published after the Spaulding review, of which 5 integrated FP services within routine HIV care and treatment (Wilcher et al. 2013). Wilcher and colleagues found that integration was positively associated with contraceptive uptake. Furthermore, suboptimal implementation in the context of health systems constraints, measured by inconsistent FP service provision and inadequate provider knowledge, may have led to the more modest effect demonstrated in the routine compared to clinical trial settings.

To inform the development of the World Health Organization (WHO) consolidated guideline on the sexual and reproductive health and rights of women living with HIV, we conducted a systematic review of the evidence on the integration of FP services into HIV care and treatment programs, building on and updating the prior systematic reviews. While there has been significant attention to the bi‐directionality of FP–HIV integration (Berer 2004; WHO et al. 2005; Farrell 2007), we focused on the integration of FP services into HIV care and treatment, a setting for which previous reviews provided limited evidence. Models to integrate FP into HIV care and treatment build on the continuity of HIV care and treatment services as a platform from which to reach women and couples living with HIV with additional services to meet their SRHR needs. We sought to 1) characterize the range of models of integration of FP into HIV care and treatment that have been evaluated, and 2) synthesize the evidence on the positive and negative outcomes of such integration.

## METHODS

### Inclusion Criteria

Studies met the inclusion criteria for the review if they 1) were published in a peer‐reviewed journal from January 1, 1990 through February 15, 2016, 2) evaluated a service delivery model that integrated FP into HIV care and treatment settings for women, 3) included a comparison HIV care and treatment setting without integrated FP services (either the same site prior to integration or a multi‐arm comparison), and 4) measured one or more of the primary or secondary outcomes specified below. We included any model of integration into HIV care and treatment settings; FP services could be provided either on‐site at an HIV clinic through a variety of service delivery models or through enhanced referrals to FP clinics. We considered a program to have enhanced referrals to FP services if one or more written policies were in place to facilitate the referral (e.g., accompanying the client to the other clinic) and/or follow up on whether the client obtained the services for which she was referred. We included studies that evaluated the integration of FP services within programs to prevent vertical HIV transmission due to the scale up of Option B+ to provide ART and other HIV care to pregnant and post‐partum women within these settings. Since the review objective is to identify FP/HIV integration models that would be feasible in real‐world conditions, we excluded interventions to provide FP within research settings. We did not restrict the search by language or location of the study.

Primary outcomes of interest for the review were unmet FP need, contraceptive use, and unintended pregnancy. Secondary outcomes were client knowledge and attitudes regarding FP for women living with HIV, client satisfaction and service quality, and costs and cost‐effectiveness. Primary outcomes were limited to women living with HIV; however, knowledge about and attitudes toward FP among men living with HIV were included, where available, given the role of partner attitudes in decisionmaking for FP.

### Search Strategy, Screening, and Data Management

We searched three electronic databases: PubMed, CINAHL (Cumulative Index to Nursing and Allied Health Literature), and EMBASE. The following terms were entered into PubMed and adapted for use in all computer databases: (“family planning” [tiab] or “contraception” [tiab] or “contraceptive” [tiab] or “birth control” [tiab] or “birth spacing” [tiab] or “reproductive counseling” [tiab]) AND (“HIV” [tiab] or “AIDS” [tiab]) AND (“integrat*” [tiab] or “linked” [tiab] or “linkage*” [tiab]). The online database search was limited to the dates January 1, 2008 through February 15, 2016; articles prior to those dates were obtained by reviewing included articles in the Spaulding et al. (2009) review that had similar, but broader, inclusion and search criteria. We also cross‐checked the included articles from four related reviews (Lindegren et al. 2012; O'Reilly et al. 2013; Wilcher et al. 2013; Medley et al. 2015). Finally, we conducted secondary reference searching on all studies included in our review to identify any remaining articles we might have missed.

Titles, abstracts, citation information, and descriptor terms of citations identified through the search were screened by two members of the study staff. Full‐text articles were obtained for all selected abstracts and screened for eligibility by two independent reviewers to determine final study selection. Differences were resolved through consensus. For each study included in the review, data were extracted using standardized forms and included information on geographic location, study design, sample size, study setting, target group, integration model, findings on process indicators and fidelity, outcomes measured, and results. Study rigor was assessed using a nine‐item tool (Kennedy et al. 2007).

## FINDINGS

**Figure 1:**
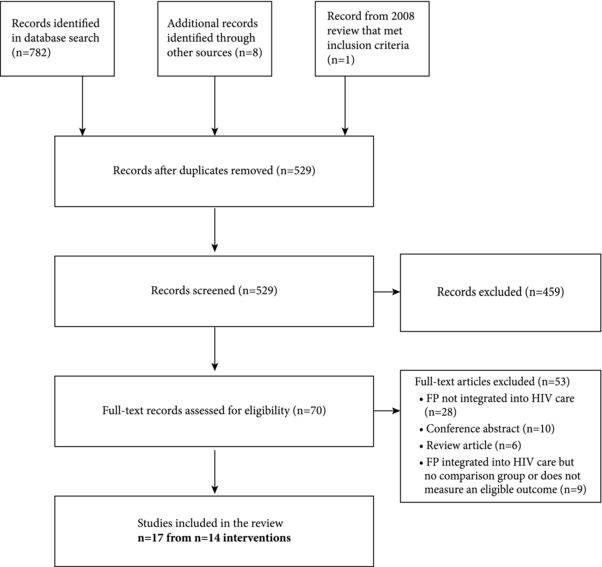
Disposition of records in the search and screening process

Our online database search yielded 782 records, and an additional 8 records were added by hand searching bibliographies (Figure 1). After removing duplicate records, 529 records were screened at the title and abstract level, resulting in 70 full‐text records evaluated. One study from the Spaulding and colleagues (2009) review of articles published prior to 2008 met the inclusion criteria and was added to the current review. Ultimately, 17 articles describing 14 distinct integrated FP–HIV programs met our inclusion criteria, 8 of which were published since the last systematic review on FP–HIV integration (Wilcher et al. 2013).

Integrated programs were evaluated in 12 countries with varying economic and socio‐political contexts. Nine studies were conducted in sub‐Saharan Africa (Chabikuli et al. 2009; Chibwesha et al. 2011; Kosgei et al. 2011; McCarraher et al. 2011; Church et al. 2012; Grossman et al. 2013; Shade et al. 2013; Baumgartner et al. 2014; Hoke et al. 2014; Sarnquist et al. 2014; Church et al. 2015; Onono et al. 2015; Wanyenze et al. 2015; Phiri et al. 2016), two in Europe (Coyne, Hawkins, and Desmond 2007; Wielding and Flynn 2015) and one in Asia (Thyda et al. 2015) (Appendix Table A1). The integration settings represent the World Bank categories for economic development: low‐income (Cambodia, Malawi, Tanzania, Uganda, and Zimbabwe), lower middle‐income (Kenya, Nigeria, Swaziland, and Zambia), upper middle‐income (South Africa), and high income (England and Scotland).

Integration settings, populations, and approaches varied. All integration models were facility‐based, primarily in HIV care and treatment settings, but also included two clinics for the prevention of perinatal HIV transmission (Hoke et al. 2014; Sarnquist et al. 2014). The settings for integration reflected the diverse levels of facilities where HIV care is delivered, ranging from small health centers to large tertiary hospitals. Outcomes were assessed among women receiving HIV care and treatment. Most studies specified a reproductive age range, two studies were limited to women receiving antiretroviral therapy (Chibwesha et al. 2011; McCarraher et al. 2011), and three studies also reported outcomes among men attending the HIV care and treatment centers (Chabikuli et al. 2009; Church et al. 2015; Wanyenze et al. 2015). One HIV care and treatment clinic served populations where many female clients had engaged in sex work (Thyda et al. 2015).

In nine of the integration models, women could obtain FP services and commodities without leaving the building where they received HIV care and treatment—what we call a “one‐stop shop” model. The one‐stop shop models varied from services provided in the same room by the same provider—used for example by an integrated HIV care and treatment clinic in Malawi and an integrated genitourinary medicine and sexual and reproductive health care center in Scotland (Wielding and Flynn 2015; Phiri et al. 2016)—to services provided in different rooms by different providers within the same clinic building (Kosgei et al. 2011). Using a different approach, four of the integration models provided a basic package of information, counseling, and condoms onsite, and referred clients to an FP center located outside the HIV clinic for all other services—what we call an “enhanced referral” model (Chabikuli et al. 2009; Chibwesha et al. 2011; McCarraher et al. 2011; Baumgartner et al. 2014). (See the intervention descriptions in Appendix Table A1 for additional detail on integration models.) The study in Swaziland was unique in comparing four clinics with varying levels of FP/HIV care and treatment integration, including two nonintegrated HIV care and treatment centers with standard referrals for FP (one of which is located within the hospital complex but in another building), and two one‐stop shop models, one providing FP in the same building as HIV care and treatment through different providers, and another providing fully integrated services in a reproductive health clinic into which HIV care and treatment was integrated (Church et al. 2012; Church et al. 2015). Therefore, this model of full integration does not exclusively serve women and men living with HIV, a significant difference from the other one‐stop shop models described in this review.

Study designs consisted of one facility‐randomized trial (Grossman et al. 2013), nine quasi‐experimental designs, and four case studies with some form of comparison group. Half of the studies relied on clinical records, audits, or routine monitoring and evaluation data for the ascertainment of outcomes and covariates, which enabled them to include a large proportion of the target population of women of reproductive age receiving HIV care.

The assessment of study rigor is presented in Appendix Table A2. All study designs included either a pre/post comparison, a control group, or a cohort. The threat of selection bias was minimized in nine studies by defining the sample using routine data as described above, or by following a systematic method to screen women attending the clinic for eligibility, most often screening everyone in attendance over the enrollment period. Six studies addressed confounding through their design or analysis, including two that randomly allocated facilities to intervention and control sites (Grossman et al. 2013; Sarnquist et al. 2014). The majority of sites in this review were nonrandomly selected to integrate FP, and these clinics may be different, and potentially better functioning in other ways that could affect contraceptive and pregnancy‐related outcomes.

Half of the reports evaluated integration within a network of clinics, providing examples of the scalability of integration. Many of the evaluations provided significant detail on implementation strategies, key decisions, and challenges, as well as reported process measures such as fidelity that would be relevant for program managers considering integration in their setting, thereby filling a knowledge gap noted previously (Spaulding et al. 2009). For example, one program at a single privately run clinic in Malawi described a phased approach that made integrating FP services feasible while adding minimal operating cost (Phiri et al. 2016).

### Information and Counseling to Support the Right to Make Reproductive Decisions

All 14 integration models provided FP information, counseling, and assessment of fertility intentions, the first component of the essential FP package (Inter‐Agency Task Team for the Prevention and Treatment of HIV Infection in Pregnant Women 2012), on‐site at the HIV care and treatment facility. While several studies reported using an informed‐choice counseling approach on contraceptive options or safer pregnancy (Chabikuli et al. 2009; Kosgei et al. 2011; McCarraher et al. 2011; Baumgartner et al. 2014; Phiri et al. 2016), most did not specify the safer conception or fertility‐services options, or, more broadly, how the programs supported the SRHR of people living with HIV.

In many integration models, female peer educators living with HIV delivered the FP information to groups, usually in the waiting room (Chibwesha et al. 2011; Grossman et al. 2013; Sarnquist et al. 2014; Thyda et al. 2015; Phiri et al. 2016). Other models added FP information to a provider checklist to cover during the clinical visit; some specified that the topic was included in individual counseling sessions; and still others did not specify how the information was provided. Many of the programs emphasized dual method use, the concurrent use of male or female condoms for barrier protection with a highly effective method of contraception, for women who wanted to prevent pregnancy (Chibwesha et al. 2011; Grossman et al. 2013; Thyda et al. 2015; Phiri et al. 2016). Some of the evaluations specified that FP information and fertility‐intention screening was intended to be part of every visit; one evaluated a three‐part peer education series to supplement general screening at visits as part of a program to prevent perinatal HIV transmission; and another evaluation emphasized screening during the peer counseling component of the ART initiation visit (Chibwesha et al. 2011). However, none of the evaluations reported the frequency with which women received more tailored or in‐depth FP information or screening for fertility intentions. In theory, ongoing assessments could better capture changes in pregnancy intentions.

### Provider Training and Supervision

The training of clinic staff or peer educators in the provision of FP services for women living with HIV was highlighted as an important component of integration in all the evaluations. Most training included both classroom‐based and practical components. For the sites that began providing long‐acting reversible contraceptive (LARC) methods, training on insertion and removal was more formalized, often involving a certification process (Sarnquist et al. 2014; Wielding and Flynn 2015). Several evaluations also explicitly described ongoing training through regular supportive supervision visits (Chabikuli et al. 2009; Baumgartner et al. 2014; Hoke et al. 2014; Phiri et al. 2016) or on‐site support by FP specialists (Thyda et al. 2015; Wielding and Flynn 2015). Some evaluations acknowledged challenges in adequately training the health providers delivering the intervention because of staff turnover or inadequate planning or budgeting to ensure that all key staff were trained, or low demand for intrauterine devices (IUDs) limiting the chance to gain practical experience in insertion and removal (Sarnquist et al. 2014). In addition to training staff from the integrated HIV care and treatment clinics, two studies that used nonintegrated HIV care and treatment settings as controls also provided training or tools to help strengthen the basic standard of care for FP information and counseling at the control sites (McCarraher et al. 2011; Grossman et al. 2013). Further, all four programs with enhanced referrals for FP commodities also provided in‐service training for clinical staff at the FP site where they referred women; other specific supports to the FP clinic included provider certification (Chibwesha et al. 2011), job aids (Chabikuli et al. 2009; McCarraher et al. 2011), and in‐service training for their supervisors, supportive supervision, and joint monthly meetings (Baumgartner et al. 2014).

### Rights‐based Family Planning Services

Among the ten integrated programs that provided effective FP methods on‐site, options included a variety of hormonal and nonhormonal methods, including male condoms, oral contraceptives, and injectable hormonal contraceptives. All but one program in Uganda (Wanyenze et al. 2015) also offered at least one LARC on‐site, IUDs or implants, and clients were referred for voluntary female or male sterilization services. Only the Malawi program reported providing female condoms (Phiri et al. 2016). The four enhanced referral sites provided only male condoms on‐site, referring women who had an interest in other methods to FP clinics (Chabikuli et al. 2009; Chibwesha et al. 2011; McCarraher et al. 2011; Baumgartner et al. 2014).

Only one of the integrated programs—the key populations clinic—explicitly described taking a rights‐based approach and minimizing coercion and stigma through its model of care, which is led by individuals living with HIV from the affected populations (Thyda et al. 2015). However, other programs noted the use of informed contraceptive choice counseling (Chabikuli et al. 2009; Kosgei et al. 2011; McCarraher et al. 2011; Baumgartner et al. 2014), the informed and voluntary use of contraception (Hoke et al. 2014), and the need to support and never dissuade women who want to become pregnant (Phiri et al. 2016). The training for one program in South Africa specified a component on the sexual and reproductive rights of people living with HIV (Hoke et al. 2014). None of the evaluations described services or referrals for infertility. There was limited detail on counseling and practical support provided for safer conception.

## OUTCOME RESULTS

Findings from each study are presented in Appendix Table A1 and are summarized below by type of outcome.

### Contraceptive Prevalence

Contraceptive prevalence was the most commonly measured outcome, reported in 12 studies, but defined in multiple ways. Modern method use was defined in most studies as including hormonal injectables, oral contraceptives, IUDs, implants, female or male sterilization, and consistent use of male or female condoms. *More effective* modern method use was a subset of the modern method definition that excluded condom use alone, which is less effective than the other modern methods in preventing pregnancy with typical use (Kost et al. 2008), despite being highly effective with correct and consistent use (National Institute of Allergy and Infectious Diseases 2001). The prevalence of dual method use, the concurrent use of male or female condoms for barrier protection with a more effective method of contraception, was measured in only a few studies. Contraceptive prevalence was reported among all women with HIV of reproductive age in some studies, and was measured among a subset of nonpregnant women who were sexually active in others.

Most programs demonstrated increases in more effective method use and/or modern method use. Among the more rigorous designs, a repeated cross‐sectional, cluster‐randomized trial in Kenya found that the odds of using a more effective contraceptive method after one year of integration relative to baseline was 1.8 times greater among women attending integrated clinics compared with those attending usual care clinics (95% confidence interval (CI): 1.2–2.6) (Grossman et al. 2013). This corresponds to an increase in more effective method use from 16.7 percent to 36.6 percent in the intervention sites, compared with 21.1 percent to 29.8 percent in the control sites over the same period. Among the evaluations with weaker designs, two studies using retrospective chart review from one‐stop model clinics serving small populations of women of reproductive age in the United Kingdom (UK) reported a shift from relying on condoms alone (decline from 30 percent to 7 percent) to more effective methods after integration (Coyne, Hawkins, and Desmond 2007), and an increase in the prevalence of more effective method use from 25 percent before integration to 39 percent after (p = 0.08), among sexually active, fertile women (Wielding and Flynn 2015). However, in two studies (Kosgei et al. 2011; McCarraher et al. 2011), the improvement in modern method use was driven by increased condom use, with no significant improvement in the use of more effective FP methods. For example, in Eldoret, Kenya the incidence of more effective FP after integration was lower in integration programs compared with usual care clinics (4.8 per 100 person‐years versus 7.8 per 100 person‐years, difference = –3.0 per 100 person‐years, 95% CI –4.6 to –1.4 per 100 person‐years), and was very low in both sites (Kosgei et al. 2011). In contrast, the uptake of condoms was significantly greater in the intervention group, leading to a significant difference in modern method initiation among women in the intervention compared to the control site (58.1 per 100 person‐years versus 44.7 per 100 person‐years). Interestingly, the evaluation reported that prior to the integration, all sites had been providing condoms and counseling on their use for transmission prevention, suggesting that messaging in an integrated setting about the effectiveness of correct and consistent condom use for the purpose of pregnancy prevention encouraged uptake.

Only three evaluations reported comparative results for dual method use, and all found potential associations in a promising direction. Dual method use increased by 16 percent among sexually active women after the start of enhanced referrals at HIV care and treatment centers in Tanzania (Baumgartner et al. 2014). The facility‐randomized trial in Kenya found that dual method use increased at integrated sites from 10.1 percent at baseline to 20.9 percent, and at usual care (control) sites from 11.5 percent to 19.1 percent. While the improvement did not differ between the integration and control sites (OR = 1.3, 95% CI 0.77–2.17), the increase in dual method use among women attending care at the control sites may be attributable to the additional training provided to all sites in this evaluation (Grossman et al. 2013). Dual method use also increased slightly, although not significantly, from 11 percent to 14.8 percent (p = 0.28) among women in Cambodia after the integration of services (Thyda et al. 2015).

### Unmet Need for Family Planning

Whereas increases in contraceptive prevalence, in the context of informed choice and voluntary use, suggest that some need for contraception has been met, it does not account for fertility intentions. Therefore, unmet need for FP, which considers fertility intentions and the ability to become pregnant, more directly measures the intended outcome of FP services for women wanting to prevent pregnancy. Only three studies evaluated the effects of integration on unmet need. An enhanced referral model in Tanzania demonstrated an 8 percent decline in unmet need after adjusting for confounders (p = 0.05,), from 25 percent to 15 percent, among sexually active women attending the 12 clinics *after* compared to *before* the integration of FP with enhanced referrals (3 percent decline among all women, p=0.10) (Baumgartner et al. 2014). A case comparison study in Uganda reported less unmet need in a clinic that provided FP services and commodities on‐site as compared with a regular care site (30.9 percent versus 45.1 percent, p < 0.01), although the statistical comparison did not account for confounders (Wanyenze et al. 2015). Another case comparison study, in Swaziland, reported the unanticipated finding of higher unmet need among women attending the most fully integrated HIV care delivery site compared with those attending the standalone HIV clinic in the same area (45 percent versus 25 percent), although the difference was not statistically significant after adjustment for confounders (aOR=2.76, 95% CI: 0.88–8.72) (Church et al. 2015).

### Unintended Pregnancy

None of the five studies evaluating pregnancy outcomes demonstrated a significant difference associated with integration. In the facility‐randomized intervention in Nyanza Province, Kenya pregnancy incidence over a one‐year period was 5.5 per 100 person‐years among the women attending the integrated sites, compared with 6.1 per 100 person‐years among the women in the control sites, a nonstatistically significant difference (Grossman et al. 2013). Likewise, in the integration program in Eldoret, Kenya no significant difference in incident pregnancy between integrated and control clinics was found (8.2 versus 7.0 per 100 person‐years, p = 0.9) (Kosgei et al. 2011). Given that an increase in desired pregnancies facilitated by FP integration is a good outcome, while an increase in unintended pregnancies indicates a poor outcome, changes in total pregnancies are hard to interpret. However, just the one small study from Scotland reported comparative data on the unplanned pregnancy rate; it declined from 5.9 to 4.1 per 100 person‐years in the pre‐ compared with the post‐integration periods, although these were based on small numbers of pregnancies and were not compared statistically (Wielding and Flynn 2015).

### Client Knowledge and Attitudes Regarding FP for Women Living with HIV

Overall, knowledge and awareness about contraceptive methods and their appropriateness for women living with HIV improved with integration. In the context of the previously described facility‐randomized trial in Kenya (Grossman et al. 2013), the average FP awareness score increased significantly between the pre‐ and post‐intervention periods among women at both the integrated and usual care sites, but awareness at endline was no higher among women attending the clinics with integrated FP compared to regular sites (Onono et al. 2015). This nondifferential improvement across sites might be attributable to the training providers received at comparison sites. Women participating in HIV care in Cambodia also had significantly improved knowledge about IUDs, injections, and male and female sterilization after integration, although knowledge of emergency contraception and female condoms remained low and there was a residual misconception among 34.5 percent of women that the IUD was not safe for women living with HIV (Thyda et al. 2015). Women participating in a program to prevent perinatal HIV transmission in Zimbabwe with exposure to the peer intervention had significantly improved knowledge of the IUD compared with the group without this exposure (85.5 percent versus 56.3 percent) (Sarnquist et al. 2014). However, not all integrated programs demonstrated improvements in knowledge. A program intended to increase access to voluntary use of LARCs and permanent methods among postpartum women who did not want to become pregnant again found that although women's knowledge that voluntary sterilization was a safe option for women with HIV improved after integration, knowledge of and attitudes toward the IUD among women living with HIV became significantly worse (Hoke et al. 2014). The authors speculated that this might have been influenced by providers’ discomfort with their own knowledge and ability to safely insert an IUD in addition to myriad implementation challenges. The increased knowledge about the safety of voluntary sterilization improved women's ability to make informed choices but did not translate into changes in potential demand, as clients were no more likely to consider undergoing sterilization in the future after integration (56.4 percent after versus 64.9 percent before, p = 0.22), indicating that factors in addition to safety were important when considering sterilization.

Attitudes also changed after FP services were introduced. In a treatment center serving key populations in Cambodia, the proportion of women living with HIV who disagreed with the statement that “people living with HIV should not have children” increased significantly from 12 percent to 26 percent, p < 0.001 (Thyda et al. 2015). Despite the improvement, the agreement among the majority of women with a statement that contravenes the reproductive choice and rights of women living with HIV suggests that the component on reproductive rights was insufficient. The evaluation of the randomized trial in Kenya found a favorable improvement in the attitude of men attending HIV care programs at the integrated sites, with a 12 percent decline in men's agreement with the statement that FP is “women's business” that was statistically significantly different from the men at the usual care sites (Onono et al. 2015). HIV care and treatment may be an opportune environment to engage men in FP.

### Client Satisfaction and Service Quality

Comparative data on client satisfaction was limited. The mixed methods case study in Swaziland compared client exit interviews among women and men attending HIV care and treatment at one of four clinics offering varying levels of integration with FP services (Church et al. 2012). The study found that overall satisfaction, including with staff demeanor, provider responsiveness, privacy, wait times, drug availability, respect for people with HIV, and protection against HIV serostatus disclosure without consent was significantly higher at the standalone HIV clinic (least integrated) than the two partially integrated sites (aOR = 0.45, 95% CI 0.25–0.81; aOR = 0.53, 95% CI 0.31–0.90), not significantly different from satisfaction with the one‐stop shop (aOR=0.84, 95% CI 0.40–1.78). While there was significant demand across all sites for more services related to FP, including STI services (48 percent), FP services (35.7 percent), counseling on sexual functioning (31 percent), and counseling on safer conception and pregnancy (29.4 percent), the demand for these services was not higher in the standalone HIV clinic (Church et al. 2015). The same evaluation found that the number of FP services received since diagnosis was not consistently higher among women attending the integrated sites, and that while women attending an integrated site were more likely to have been counseled on FP and safe pregnancy, they were no less likely to have unmet need for contraception. They were, however, less likely to have received condoms than women attending the HIV standalone clinic, where condoms have been a mainstay of programs to prevent HIV transmission. Data are needed from more programs on the access, quality, and cost of FP services from the perspective of women attending care and treatment in HIV care and treatment settings with, compared to without, integrated FP services.

### Costs and Cost‐effectiveness

Cost data were available only for the facility‐randomized trial in Kenya. The estimated cost of delivering FP services was $14.10 per woman attending the clinic with a one‐stop model, compared with $7.03 per woman attending a nonintegrated site, within the range of previously published costs ($6 to $113) (Shade et al. 2013). Linking to outcome data, the cost per additional use of more effective family planning was $70.93 in the one‐stop model compared with $80.62 in the nonintegrated site, for a marginal cost efficiency ratio of $65.39. The cost‐effectiveness analysis estimated a cost of $1,368 per pregnancy averted. Furthermore, the authors identified economies of scale for integration in clinics serving larger populations, with the cost of integration declining from an additional $9 per woman in a clinic serving 200 women living with HIV to a cost‐savings of $0.48 per woman in a clinic serving 800 women.

## DISCUSSION

Overall, the integration of FP into HIV care and treatment settings was associated with higher levels of modern method contraceptive use and knowledge among women living with HIV. The diversity of integration models and their successful implementation across a range of settings suggests that such models are feasible. Between 48 percent and 57 percent of women living with HIV are engaged in HIV care and treatment, making it a relevant platform from which to empower women to make informed decisions regarding their SRHR; this coverage is expected to improve further with progress toward the UNAIDS 90‐90‐90 goals for HIV testing, treatment, and effective viral suppression developed at the 21st International AIDS Conference (UNAIDS 2014). The effectiveness of the modern methods chosen differed by setting, however, with some improvements driven by higher condom use alone, whereas other sites demonstrated significant improvements in the use of LARCs or other highly effective methods. In many evaluations, the level of more effective FP use and dual method use remained low, even when it was higher relative to comparison groups. More effective FP use (excluding condom use alone) remained below 20 percent in some settings.

Evidence for changes in unmet need for FP was more limited, although two of the three evaluations that measured unmet need suggested possible improvements (Baumgartner et al. 2014; Wanyenze et al. 2015). However, the level of unmet need was extremely high, even at the integrated sites, and points to the need for other interventions to reduce the gap between pregnancy intentions and contraceptive use. There was insufficient data to evaluate the effect of FP/HIV integration on unintended pregnancy. Integrated services improved the important intermediate outcomes of women's knowledge of the benefits and risks of contraceptive methods for women with HIV, but not uniformly, with some sites reporting mixed or negative results. More research to understand variation in knowledge around specific types of contraception is recommended to ensure that rights to full information on the range of contraceptive choices are upheld and that provider training is sufficient.

Commonalities emerged across programs, including the use of peer educators to deliver information about FP for women and couples living with HIV. Task‐sharing of provision of information on contraception and safer pregnancy between clinicians and peer educators could lead to more efficient use of clinicians’ time, and the messages delivered by peer educators could be expanded to incorporate SRHR and sexuality education around coercion in sexual and reproductive health decisionmaking. Most one‐stop sites offered a good range of contraceptive choices on‐site, including short‐ and long‐acting methods, hormonal and nonhormonal methods, and condoms for dual protection. The female condom and emergency contraception were not available through most integrated programs, confirming a limitation noted by a previous review (MacCarthy et al. 2012).

High‐quality training in FP for health providers, involving both didactic learning and practical applications, was consistently identified as a key factor in the success of integration. Two particular issues that emerged across settings as barriers to the uptake of LARCs were providers’ limited confidence in their ability to insert and remove IUDs as well as their knowledge of the safety of various methods for women living with HIV. Although decentralized training by traveling trainers at the health facilities has the potential benefit of reaching more staff with potentially fewer service disruptions, the low baseline demand for the IUD in some settings makes it difficult for the providers to gain the practical experience needed to offer them to women reliably or to complete certification. In some settings, bringing providers into hub facilities that have higher demand for the IUD for practical training could help build their proficiency before they return to sites where initial demand is low but may increase with improved availability and quality.

All of the included studies focused more on FP services and outcomes among women wanting to prevent unintended pregnancy than among women who wanted to become pregnant. Using a comprehensive definition of FP, the completed referrals to programs to prevent perinatal transmission could be one process indicator of successful linkage for women who would like to become pregnant, for example. There is a small but growing literature on safer conception and assisted reproductive technologies for women and couples living with HIV (Schwartz et al. 2014; Goggin et al. 2015; Heffron et al. 2015; Steiner et al. 2016), with data from implemented programs being even more limited. The recent global survey reported that the majority of women with HIV had received advice on safe conception, but only half had received practical support for safe conception (Salamander Trust 2014), and such coverage is likely lower among women from low‐ and middle‐income countries. Therefore, it is possible that women are benefiting from such programming, although they were not measured as outcomes of the integrated FP services. Finally, input and leadership from women living with HIV have illuminated critical gaps in the delivery of FP services available in many settings, including attitudes and practices among health workers that do not support and in some cases violate the SRHR of women and couples living with HIV (WHO and Harvard School of Public Health 2010). The view that HIV infection and safe pregnancy were often perceived as mutually exclusive before widespread availability of ART is still reflected in the attitudes and biases of some antenatal service providers treating women living with HIV, although these attitudes were not assessed by most of the evaluations. Measures must be taken to ensure that the SRHR of women living with HIV are respected through gaining information about the full range of contraceptive options and being supported to make a fully informed, voluntary choice.

There are several limitations to the evidence available. Overall, the study designs and analyses that met our inclusion criteria did not adequately adjust for confounding and bias, and the majority of comparisons were of crude differences. In addition, few studies measured unmet need or unintended pregnancies because of lack of data on fertility desires, and several studies used nonstandard definitions and/or substandard ascertainment of unintended pregnancy. Due to wide variations in the consistency of condom use and social desirability bias in reporting, changes in contraceptive use measures are most useful when they disaggregate and compare the relative contribution of condom‐only, dual method use, and more effective method use‐only to modern method use. While our review did not explicitly include outcomes among women and couples within serodiscordant relationships who were accessing integrated FP services at HIV care and treatment centers through the partner living with HIV, our search would have likely identified them. Finally, as with all reviews, the bias toward publishing positive over null or negative evaluation results may lead to the inclusion of programs that are more likely to have positive outcomes, relative to all integrated programs.

## CONCLUSION

A body of evidence supports the feasibility, benefits, and potential cost‐effectiveness of integrating FP into HIV care and treatment services in a range of settings. However, the quality of the evidence of effectiveness is modest. Furthermore, some integration efforts demonstrate that improving access is not sufficient to improve the uptake of more effective contraceptive methods among women living with HIV who want to avoid pregnancy. Integration, particularly in contexts where the use of effective contraception is low, must address the cultural and gender norms that impact women's decisionmaking regarding FP alongside improving access to FP information and services within routine HIV care.

## Appendix

**Table A1:** Integration models, outcomes

**Citation, location**	**Study design, sample size**	**Study setting, target group**	**Description of FP integration**	**Outcomes**
Baumgartner et al. 2014 **Location**: Iringa and Morogoro Regions, Tanzania	**Design**: Repeated cross‐sectional pre‐ and post‐test **Sample size**: n=323 women pre‐intervention; n=299 women post‐intervention	12 public HIV care and treatment centers (CTCs): 6 hospitals, 6 health centers purposively selected for having high CTC client load and a co‐located FP clinic within the same facility **Target group**: Women of reproductive age living with HIV attending HIV care and treatment	**Enhanced referral**: Piloting a facilitated referral model for integrating FP services into CTCs managed through the TUNAJALI program. The 7 steps of the referral model included: 1) Screen clients for unintended pregnancy risk, 2) Provide informed choice counseling on contraceptive options or safer pregnancy, 3) Refer for services, if desired, using the CTC referral form, 4) Document the referral, 5) Staff member accompanies the client to the FP clinic, 6) CTC client accesses FP services in a timely manner, and 7) CTC and FP staff follow up on referrals and services through monthly meetings and tracking of completed referrals. The screening process was supposed to be initiated at every clinical visit for women of reproductive age. **Training**: For CTC and FP providers and supervisors, followed by supportive supervision for CTC and FP staff, and monthly meetings with FP and CTC staff to review progress **Additional components**: FP group education sessions, monitoring using national forms and registers; job aids.	**Unmet need**: 3% decline in unmet need post‐intervention among all women (p=0.10). 8% decline in unmet need among sexually active women (p=0.05). **Contraceptive use**: 12% increase in contraceptive use among sexually active women (p=0.01). 16% increase in dual‐method use (p<0.01).
Chabikuli et al. 2009 **Location**: Nigeria	**Design**: Retrospective cohort comparing the pre‐ and post‐intervention periods using aggregate sex‐stratified health services data **Sample size**: n=71 facilities	71 public health facilities providing HIV treatment, prevention of perinatal transmission, and/or HIV testing and counseling (64 with treatment and FP) involved in a facilitated referral model between HIV and FP units **Target group**: Women living with HIV attending HIV care and treatment	**Enhanced referral**: Piloting a facilitated referral model for integrating FP services into 71 public health facilities providing HIV treatment, prevention of perinatal transmission, and/or HIV testing and counseling (64 facilities with ART and FP) **Training**: For providers, followed by supportive supervision, 4 job aids (at the FP clinics), 1 job aid at the ART clinic **Additional components**: Adding HIV data elements to the FP register and improving data flow from the facility to the state and federal level	**Contraceptive use**: Monthly mean couple years of protection delivered at the FP centers increased from 32.3 before integration to 38.2 after integration (p<0.001)(*data includes HIV‐ and HIV+) Service ratio of referrals from HIV treatment centers increased by 3.4% after integration, and 81.7% of these referrals were women living with HIV
Chibwesha et al. 2011 **Location**: Lusaka, Zambia	**Design**: Cohort, one year follow‐up post‐ integration, November 2009–November 2010) **Sample size**: n=18,407 women	16 primary‐care HIV clinics **Target group**: Women of reproductive age living with HIV on ART attending HIV clinics	**Enhanced referral**: Peer counselor–delivered standardized FP counseling, focused on promotion of dual‐method use. A printed counseling tool was used as an aid to provide information on barrier methods, short‐ and long‐acting reversible contraception, and sterilization. Clients were referred to FP clinics for all commodities. **Training**: 109 peer counselors were trained to provide FP counseling, with a focus on dual‐method use. In addition, the study trained and certified 42 FP nurses at separate FP departments located within the same facilities where clients would be referred.	**Contraceptive use**: Only 9.8% of the women not using modern contraception at baseline desired a referral for contraceptives at the FP clinic. Contraceptive uptake was demonstrated for 61.6% of the women desiring a referral to FP. At baseline, the prevalence of modern contraceptive use was 59.2%, with 75% reporting condom use, followed by DMPA (9.9%), oral contraceptives (6.7%), implants (5.4%); IUDs (3.4%); and prior sterilization (1.1%). Among those using modern contraception, only 17.7% reported dual‐method use and 26.5% reported more effective (noncondom) FP use.
Church et al. 2012; Church et al. 2015 **Location**: Manzini, Swaziland	**Design**: Cross‐sectional, mixed methods comparative study of 4 clinics with different integration models **Sample size for quantitative questionnaire**: n=602 women and men attending HIV care **Sample size for qualitative interview**: n=16 providers; n=22 women and men on ART	4 HIV clinics with different models of care **Target group**: Women living with HIV engaged in HIV care	**One‐Stop Shop: (**Clinic A)—All reproductive health (RH) and HIV services are intended to be available from a single provider in a single location; clinic formerly provided RH care before HIV services were integrated. Clinic B (partially integrated)—HIV treatment services decentralized in a primary‐care clinic. RH and HIV services are intended to be offered by different providers in different locations, but within the same building Clinic C (partially standalone)—HIV unit in a separate building on the grounds of a facility that offers RH services in other buildings; operates through referrals Clinic D (fully standalone HIV clinic)—HIV clinic, FP counseling, and condoms are available onsite, but must get referral for contraceptives **Training**: Providers at the HIV standalone clinic had been trained on RH counseling	**Unmet need**: Unmet need was higher in the most integrated model, Clinic A, compared with the HIV standalone clinic, Clinic D, (45% versus 25%); the difference was not statistically significant after adjustment for confounders **Contraceptive use**: Since diagnosis, clients in the HIV standalone clinic, Clinic D, reported less FP counseling but no difference in FP method provision. Condom provision was significantly higher in the standalone clinic, Clinic D. ***Secondary*** **Client satisfaction**: There was significant demand for more FP services: across sites, 48% would like STI services, 35.7% would like FP services, 31% would like counseling on sexual functioning, and 29.4% would like counseling on how and when to get pregnant. The demand for these additional services was no higher in the standalone HIV clinic than in the more integrated sites. Demand was greatest in the partially integrated sites. **Number of FP services received since diagnosis**: The proportion of clients reporting FP services since HIV diagnosis varied by clinic, but was not consistently higher in the integrated sites. FP counseling was less common in the HIV standalone clinic, but FP method provision was not different and condom provision was significantly higher in the HIV standalone clinic.
Coyne, Hawkins, and Desmond 2007 **Location**: Slough, United Kingdom	**Design**: Serial cross‐sectional **Sample size**: n=60 *Time 1*: n=30 women *Time 2*: n=30 women	1 integrated center for Genitourinary Medicine (GUM) and SRH services **Target group**: Women of reproductive age living with HIV starting HIV care	**One‐stop shop**: The Garden Clinic started a specific clinic (FP Plus) to provide HIV‐positive women clients with screening for STIs, contraception, pre‐conception counseling, and cervical cytology. Commodities included LARC methods. The Garden Clinic already worked on a model of integrated sexual health care, and FP Plus was staffed by doctors and senior nurses trained in both STI management and FP.	**Contraceptive use**: The proportion of women who relied on condoms alone for contraception declined from 30% to 7% before versus after integration (no statistical test performed). In the post‐integration period, 6 (20%) of the women initiated highly effective contraceptive methods at the FP Plus Clinic (n=3 IUD and n=3 DMPA or implant).
Grossman et al. 2013; Shade et al. 2013; Onono et al. 2015 **Location**: Nyanza Province, Kenya	**Design**: Repeated cross‐sectional, facility‐randomized trial **Sample size**: n=12 intervention clinics with n=2,593 person‐visits (from 1,684 women) in the baseline period; n=6,972 person‐visits in the endline period n=6 control clinics with n=3,089 person‐visits (from 1,900 women) in the baseline period; n=5,559 person‐visits in the endline period	18 public HIV clinics **Target group**: Women of reproductive age living with HIV attending HIV treatment	**One‐stop shop**: Clinics were randomized to integrate FP services into the HIV clinic (n=12 sites) or to refer clients to FP clinics within the same facility (n=6). The integrated sites provided a method mix including LARC methods. All sites provided FP information and condoms. **Training**: Peer educators were trained in leading group education. At all sites, staff were trained to provide reproductive health counseling through in‐class and practical components. Regular refresher trainings were held.	**Contraceptive use**: Highly effective contraceptive prevalence increased from 16.7% to 36.6% in the intervention clinics compared with 21.1% to 29.8% in the control sites over the same period (odds ratio (OR)=1.81, 95% confidence interval (CI): 1.24, 2.63) **Pregnancy**: Incident pregnancy within the first year after integration was 1.5 in the intervention group compared with 1.7 in the comparison group, but there was no statistically significant difference (IRR=0.90, 95% CI: 0.68, 1.20). NB: The study did not have data on pregnancy intention. ***Secondary*** **Cost‐effectiveness**: The marginal cost per additional woman using more effective contraception was $65. The marginal cost was $1,368 for each pregnancy averted. Economies of scale were identified in clinics serving larger populations (Shade et al. 2013). **Client knowledge and attitudes toward FP**: Mean FP awareness scores increased between the pre‐ and post‐intervention periods among clients at both the intervention and control sites; there was no difference in awareness of methods between the integrated and non‐integrated clinics at endline (Onono et al. 2015). **Client attitudes toward male involvement in FP**: In the intervention sites, the proportion of males that agreed with the statement that FP is “women's business” declined by 12% after the intervention, a more significant decline than seen at the control sites, aOR=0.43, (95% CI: 0.22, 0.85) (Onono et al. 2015).
Hoke et al. 2014 **Location**: Cape Town, South Africa	**Design**: Repeated cross‐sectional pre‐ and post‐test **Sample size**: n=265 women pre‐intervention; n=266 women post‐intervention	5 public maternal and child health services in low‐income, peri‐urban areas **Target group**: Women living with HIV who are ≤ 6 months postpartum, attending child health services	**One‐stop shop**: Providers were trained on reproductive health for women living with HIV, with a focus on IUD information, insertion and removal, and sterilization for women desiring no further children. The intervention included an improved referral system for tubal ligation. **Training**: Training on RH, IUD, and sterilization. Followed by job aids for reproductive health counseling, and twice‐monthly mentoring and coaching.	**Contraceptive use**: Proportion using modern contraception did not improve between the pre‐ and post‐intervention periods (89.8% pre versus 83.5% post, p=0.03). Use did not increase for IUD (0% pre versus 0.5% post; p=0.48) or sterilization (7.1% pre versus 8.6% post; p=0.57). Majority (>70%) at both periods used an injectable hormonal contraceptive. ***Secondary*** **Client knowledge and attitudes toward FP**: The percent counseled on IUDs increased significantly from 7.8% to 23.7%; however, IUD knowledge was lower after the intervention. Post‐intervention, a greater proportion of postpartum women living with HIV reported correct knowledge of sterilization as a contraceptive option, although fewer women reported that they would consider it in the future (56.4% post versus 64.9% pre; p=0.22)
Kosgei et al. 2011 **Location**: Eldoret, Kenya	**Design**: Retrospective cohort study **Sample size**: n=1,453 women under intervention care team, n=2,578 women under regular care team	Large HIV clinic located within the Moi Teaching and Referral Hospital **Target group**: Women of reproductive age living with HIV attending HIV treatment	**One‐stop shop**: The medical practice for Clinical Team I added a private room for Reproductive Health. Family planning services provided by nurses with FP experience in the RH room, while original clinical staff continued to serve HIV‐related needs.	**Contraceptive use**: Incidence of modern method use was greater in the integrated site (58.1 per 100 person years (py) versus 44.7 per 100py; incidence rate difference (IRD)=16.4, 95% CI: 11.9, 21.0), as was reported new condom use (53.4 per 100 py versus 36.9 per 100 py; IRD=13.5, 95% CI: 8.7, 18.3). Incidence of noncondom FP use was low, and was lower in the intervention site, however (4.8 per 100 py versus 7.8 per 100 py; IRD=–3.0, 95% CI: –4.6, –1.4). **Pregnancy**: There was no significant difference in the incidence of pregnancy between the integrated and regular sites (8.2 versus 7.0 per 100 py; IRD=1.2, 95% CI: –0.6, 3.0). NB: The study did not have data on pregnancy intention.
McCarraher et al. 2011 **Location**: Cross River State, Nigeria	**Design**: Quasi‐experimental, pre‐post with comparison group **Sample size**: n=335 women pre‐intervention; n=274 with post‐intervention data	Secondary‐ and primary‐level health care facilities in 5 local government areas (LGAs) **Target group**: Women of reproductive age living with HIV on ART	**Enhanced referral**: Expanded a facilitated referral model for integrating FP services into HIV treatment services in 5 local government areas (see Chabikuli et al. 2009, for original model). **Training**: For providers, supportive supervision, 4 job aids (at the FP clinics), 1 job aid at the ART clinic	**Contraceptive use**: Modern method use increased in both the intervention (from 21.8% to 34.7%) and the control (5.3% to 17.6%) sites; there was no significant intervention effect (p=0.94). The use of highly effective contraceptives increased from 7% to 12% in the intervention sites and from 4% to 6% in the basic sites; the difference by integrated services was not assessed. **Pregnancy**: Reported 31 pregnancies between baseline and follow‐up, 23 in the enhanced referral sites and 8 in the basic group. 5/31 of the pregnancies were among women who initially reported wanting no more children, while 22/31 were wanted pregnancies and 4/31 were among women who were unsure. There was no adjusted comparison of the outcome by integration status.
Phiri et al. 2016 **Location**: Lilongwe, Malawi	**Design**: Cross‐sectional comparison of routine data from an HIV clinic with FP integration and a similar HIV clinic that refers to an FP clinic on the same facility grounds **Sample size**: Not provided	2 public HIV clinics run by Lighthouse Trust **Target group**: Women living with HIV of reproductive age attending HIV treatment	**One‐stop shop**: Stepwise integration of FP services into Lighthouse Clinic for HIV treatment and care. Services included FP IEC (group‐level information in the waiting rooms delivered by peer educators), counseling, contraceptive commodities, and later, cervical screening. FP commodities expanded over time to include LARC methods. **Training**: Providers received didactic and practical training and supportive supervision **Additional components**: The integration involved renovation of the clinic to create private rooms for counseling and gynecologic exams and clinic supplies. Protocols were developed for providing the new services and the routine electronic data system was expanded to capture new services. The clinic became an official MOH point for FP service delivery.	**Contraceptive use**: Contraceptive prevalence among nonpregnant women of reproductive age was 42% at the intervention site compared to 29% at the site with referrals. At the intervention clinic, 859 women started modern FP, made up of DMPA (55%), IUD (19%), combined oral contraceptives (14%), and implants (12%).
Sarnquist et al. 2014 **Location**: Chitungwiza, Zimbabwe	**Design**: Quasi‐experimental prospective study with control group **Sample size**: n=65 women in intervention; n=33 women in standard of care	4 public polyclinics (ANC and MCH) **Target group**: Women living with HIV seeking ANC between 26 and 38 weeks gestation	**One‐stop shop**: During the intervention period, women participated in 3 peer‐led, 90‐minute group education sessions on sexual negotiation skills, empowerment, FP, and communications. Providers were trained in FP as described below. Contraceptives, including LARC, were provided on‐site. **Training**: Nurses at the clinics participated in a 5‐day training in FP by national FP commission trainers, including the insertion and removal of LARC. Training included clinical/practical component.	**Contraceptive use**: LARC prevalence was very high in both groups by 3 months postpartum (87.1% among women in the intervention group, compared with 81.8% in the control; p=0.34). Prior to most recent pregnancy, ever use of LARC was 2% among women in the intervention group, and 0% in the control group; therefore the uptake was significant (p=0.01), although it did not differ by exposure to the intervention. ***Secondary*** **Client knowledge and attitudes toward LARC**: Knowledge of IUD as a highly effective method was significantly higher in the group that had the peer intervention, 85.5% versus 56.3% (p<0.01).
Thyda et al. 2015 **Location**: Phnom Penh, Cambodia	**Design**: Pre‐ and post‐test **Sample size**: n=250 pre‐intervention; n=249 post‐intervention	1 HIV care clinic serving key populations **Target group**: Women of reproductive age living with HIV who had at one time been involved in sex work attending routine HIV care	**One‐stop shop**: The Chhouk Sar (CS) clinic is a peer‐managed clinic that provides HIV care for key populations. The clinic began providing FP services and commodities on‐site. Method mix included LARC. Women received referrals for sterilization services. FP services were free of charge to the client and available 5 days per week. IEC and screening for unmet need was provided by the peer staff in the waiting room, and only clients with identified needs were referred to a private counseling room for individual counseling with a midwife or doctor (internal referral). Messages emphasized dual‐method use. IEC was supported by job aids including flip charts. **Training**: Staff from Marie Stopes International Cambodia trained clinic staff during the first 6 months of the intervention, including theoretical and practical components. It appears that the trainers provided extended on‐site observation and supportive supervision for 6 months. The 2 fully trained health providers then began providing FP services without supervision.	**Contraceptive use**: There was no significant change in the use of an effective FP method (12.6% before versus 16.4% after, p=0.86) or the proportion of women reporting dual‐method use (11% before versus 14.8% after, p=0.28); 76.2% of women continued to rely solely on condom use for contraception after the intervention. ***Secondary*** **Client knowledge and attitudes toward contraception**: Client awareness of modern contraceptives was higher after integration for IUD (96.8% versus 92%, p<0.05), injections (77.5% versus 67.2%, p<0.05), implants (85.5% versus 71.6%, p<0.01), and male sterilization (14.8% versus 3.6%, p<0.01) and female sterilization (49.4% versus 22.4%, <0.01), although not for emergency contraceptives or female condoms. Client knowledge of FP improved significantly regarding safety of FP use and specifically IUD use by women with HIV. After integration, 79.5% of the women using condoms only reported that they believe that the condoms afford them sufficient protection from pregnancy, a significantly higher proportion than baseline. **Attitudes**: 26.5% after versus 12% before integration disagreed with the statement, “PLHIV should not have children.” The proportion of women who planned to use FP in the future was 94.3% after integration compared with 60.9% before integration (p<0.001).
Wanyenze et al. 2015 **Location**: Kampala, Uganda	**Design**: Cross‐sectional comparing one‐stop shop integrated facility and site with information but no contraceptive provision **Sample size**: n=797: n=389, intervention; n=408, regular care	2 HIV care clinics, one within the national teaching hospital, one within a private faith‐based hospital **Target group**: Women of reproductive age living with HIV who are sexually active and engaged in HIV care	**One‐stop shop**: The Mulago HIV clinic, based in the national teaching hospital, provides FP education to groups and individualized counseling. Condoms, pills, and injectables are available on‐site. Clients are referred to the FP clinic within the same larger hospital campus to access LARC and tubal ligation.	**Unmet need**: 30.9% in the integrated site compared with 45.1% in the regular care site (p<0.01) **Contraceptive use**: Use of an effective FP method (includes reported consistent condom use) was higher at the intervention site (57.9% versus 50.0%; p=0.04); no difference in the use of any FP method (or any modern method (includes any condom use). **Pregnancy**: 16% of the incident pregnancies were among women who reported at baseline that they did not want more children.
Wielding and Flynn 2015 **Location**: Edinburgh, Scotland	**Design**: Cohort using routine data and clinical case notes before and after the integration of the SRH and Genitourinary Medicine (GUM) services **Sample size**: n=68 pre‐integration; n=74 post‐integration	The NHS Lothian Integrated Center for GUM and SRH **Target group**: Women living with HIV of reproductive age engaged in routine HIV care	**One‐stop shop**: A purpose‐built integrated center that combined previously standalone GUM and SRH clinic services into a single one‐stop location. In addition to the usual GUM services, which included HIV treatment and routine care, the integrated services included FP counseling and provision, and cervical cancer screening. **Training**: Prior to integration, medical staff were trained in contraceptive counseling, provision, and cervical screening. Clinicians earned certification for contraceptive implant fitting through the Faculty of Sexual and Reproductive Healthcare's letter of compliance. All physicians were certified in fitting IUDs. Supportive supervision was provided by senior physician cover for GUM and SRH, and, when needed, the clinicians could refer clients to other specialist SRH services that were provided on‐site.	**Contraceptive use**: Use of an effective contraceptive method by fertile, sexually active women was 24.9% pre‐integration and 39.3% post‐integration (p=0.07). The prevalence of LARC increased from 16.1% to 29.8% (p=0.07). **Unintended pregnancy**: The unplanned pregnancy rate was 5.9 per 100 py in the pre‐intervention period and 4.1 per 100 py in the post‐intervention period. Statistical significance of the change was not reported.

**Table A2:** Study rigor assessment

Citation	Study design includes pre/post intervention data	Study design includes control or comparison group	Study design includes cohort	Comparison groups equivalent at baseline on socio‐demographics	Comparison groups equivalent at baseline on outcome measures	Selection process minimizes bias^a^	Participants randomly allocated to the intervention	Control for potential confounders	Follow‐up rate ≥75%
Baumgartner et al. 2014	Yes	No	No	N/A	N/A	Yes	No	Yes	N/A
Chabikuli et al. 2009	Yes	No	No	N/A	N/A	Yes	No	No	N/A
Chibwesha et al. 2011	No	No	Yes	N/A	N/A	No	No	No	NR
Church et al. 2012; Church et al. 2015	No	Yes	No	N/A	N/A	Yes	No	Yes	N/A
Coyne, Hawkins, and Desmond 2007	Yes	Yes	No	N/A	N/A	Yes	No	No	N/A
Grossman et al. 2013; also Shade et al. 2013 and Onono et al. 2015	Yes	Yes	No	Yes	No	Yes	Yes	Yes	N/A
Hoke et al. 2014	Yes	No	No	N/A	N/A	Yes	No	No	N/A
Kosgei et al. 2011	No	Yes	Yes	Yes	No	No	No	Yes	Yes
McCarraher et al. 2011	Yes	Yes	Yes	No	No	No	No	Yes	Yes
Phiri et al. 2016	No	Yes	No	NR	NR	No	No	No	N/A
Sarnquist et al. 2014	No	Yes	Yes	Yes	Yes	Yes	Yes	No	Yes
Thyda et al. 2015	Yes	No	No	N/A	N/A	NR	No	No	N/A
Wanyenze et al. 2015	No	Yes	No	No	N/A	Yes	No	Yes	N/A
Wielding and Flynn 2015	Yes	No	No	NR	NR	Yes	No	No	N/A

N/A = Not applicable. NR = Not reported.

a For most studies, integration is a facility‐level intervention, and the target populations for the included studies were women living with HIV who were attending HIV treatment. Therefore randomization was not superior to other approaches to reduce selection bias, including using a census of all visits (e.g., the studies using electronic medical data) or eligibility screening of all or a randomly selected subset of women attending the sites during an enrollment period. Studies using either of these approaches for selection of the study sample were assessed as having minimized selection bias.
